# Safety of Antiandrogens for the Treatment of Female Androgenetic Alopecia with Respect to Gynecologic Malignancies

**DOI:** 10.3390/jcm13113052

**Published:** 2024-05-23

**Authors:** S Morteza Seyed Jafari, Kristine Heidemeyer, Robert E. Hunger, Pierre A. de Viragh

**Affiliations:** Department of Dermatology, Inselspital, Bern University Hospital, 3010 Bern, Switzerland

**Keywords:** 5α-reductase inhibitors, androgenetic alopecia, cyproterone acetate, dutasteride, finasteride, gynecologic malignancies, safety, spironolactone

## Abstract

The most common type of alopecia in women is female androgenetic alopecia (FAGA), characterized by progressive hair loss in a patterned distribution. Many oral therapies, including spironolactone (an aldosterone antagonist), androgen receptor blockers (e.g., flutamide/bicalutamide), 5-alpha-reductase inhibitors (e.g., finasteride/dutasteride), and oral contraceptives, target the mechanism of androgen conversion and binding to its respective receptor and therefore could be administered for the treatment of FAGA. Despite significant advances in the oral treatment of FAGA, its management in patients with a history of gynecological malignancies, the most common cancers in women worldwide, may still be a concern. In this review, we focus on the safety of antiandrogens for the treatment of FAGA patients. For this purpose, a targeted literature review was conducted on PubMed, utilizing the relevant search terms. To sum up, spironolactone seems to be safe for the systemic treatment of FAGA, even in high-risk populations. However, a general uncertainty remains regarding the safety of other medications in patients with a history of gynecologic malignancies, and further studies are needed to evaluate their long-term safety in patients with FAGA and risk factors to establish an optimal risk assessment and treatment selection protocol.

## 1. Introduction

The most common type of alopecia in women is female pattern hair loss or female androgenetic alopecia (FAGA) [1]. The condition can begin at any age around and after puberty [1], with up to 40% of women developing FAGA by the age of 70 or older. Patients often experience psychological distress as their hair becomes thinner, and therefore seek treatment to stop future hair loss and stimulate regrowth [1].

The pathomechanism of FAGA is not fully understood but its etiology is most likely multifactorial and polygenic, defining an individual’s susceptibility to androgen action on the hair [2]. Studies on inheritance show that women with male or female first-degree relatives with androgenetic alopecia have incidence rates of 54% and 21%, respectively [2,3]. Dihydrotestosterone (DHT) has been shown to set off FAGA by shrinking terminal hair follicles into vellus hair follicles, while estrogen promotes hair development and controls hair loss [4,5,6,7]. Sex hormone-binding globulin (SHBG), which is the major transport protein for circulating estradiol and testosterone, may also play a role [4]. Elevated levels of androgens inhibit the synthesis of SHBG, whereas estrogen stimulates it [4]. Testosterone is typically bound to SHBG in the serum [4,8]. Only free testosterone and estradiol are active in peripheral tissues, so SHBG levels have been shown to correlate inversely with the severity of FAGA [4,9]. Still, the causal association between FAGA and androgens is still an area of debate. Although FAGA has classically been described as a sign of hyperandrogenism and a subset of the patients do have hormonal dysregulation [2,10], the majority of women with FAGA neither have excessive levels of androgens nor exhibit any symptoms or signs of abnormal androgen levels [9,10,11]. FAGA has also been documented in individuals diagnosed with hypopituitarism or complete androgen insensitivity syndrome, where androgen levels are undetectable [11,12,13].

Many oral therapies, including androgen receptor blockers, 5-alpha-reductase inhibitors, and oral contraceptives, target androgen synthesis, conversion, or binding to its receptor [11,14] (Table 1). Spironolactone, an aldosterone antagonist and a potassium-sparing diuretic, prevents testosterone and DHT from interacting with intracellular androgen receptors [11,14]. Furthermore, it can weakly inhibit androgen synthesis [11,15]. The 5-alpha-reductase inhibitors (e.g., dutasteride and finasteride) prevent the conversion of testosterone to DHT, leaving androgens that do not bind securely to their receptors [11]. Cyproterone acetate (CPA) similarly competitively prevents DHT from attaching to its receptors [11,16]. It is a progestogen that reduces testosterone levels by suppressing the release of gonadotropines through pituitary-mediated suppression [11,16]. Flutamide and bicalutamide are further oral antiandrogens that inhibit the androgen absorption and its nuclear binding in target tissues [11,16].

Despite advances in the oral treatment of FAGA, such as oral minoxidil as a non-specific stimulator, its management in patients with a history of breast cancer and gynecological malignancies, the most common cancers in women worldwide, may still be a concern. In this review, we focus on the safety of antiandrogens for the treatment of FAGA in such patients.

## 2. Materials and Methods

A targeted literature review was conducted on PubMed, utilizing the following search terms: 5α-reductase inhibitors, Androgenetic alopecia, Antiandrogens, Bicalutamide, Breast Cancer, Cyproterone acetate, Dutasteride, Finasteride, Flutamide, Gynecologic malignancies, and Spironolactone. Papers were selected based on their relevance to the topic, as determined by title and abstract screening. The review included studies in English that primarily focused on single cases, case series, and both retro- and prospective trials, with no time restrictions applied. The findings are succinctly presented in the form of a narrative review, offering a comprehensive summary of the key issues. For ease of reference, Table 2 provides an organized overview of the discussed publications in chronological order.

## 3. Spironolactone

Spironolactone is an aldosterone antagonist diuretic with structural similarity to progesterone [26]. Although it can bind to both the estrogen and progestin receptors, its predominant affinity is for the androgen receptor [26]. Spironolactone is the most commonly used off-label antiandrogen, a well-tolerated therapy for the management of FAGA, hirsutism, and acne, even if studies on its efficacy are limited [37,38]. While spironolactone has a well-established long-term safety profile, there is a theoretical risk that spironolactone may promote certain cancers, mainly breast and uterine cancer, due to its antiandrogenic and progesterone effects and its potential stimulation of the breast (tenderness in women and gynecomastia in men), secondary to altering the balance between androgens and estrogen [25,26,28,31]. Thus, many physicians avoid prescribing it to patients with a history of or at high risk of breast cancer, and because of old publications raising concerns that the drug may increase the risk of breast cancer [25,39,40]. Spironolactone also carries an official FDA warning regarding possible tumorigenicity [33]. However, this warning is based on animal studies using doses up to 150 times higher than human doses that found the development of testicular, liver, and breast adenomas [33,41]. Notwithstanding, the International Agency for Cancer Research has not classified spironolactone as a carcinogen [42].

A case-control study in the United States found a positive association between treated hypertension, diuretic use, and breast cancer risk in women aged 50–75 years [22]. However, the use of spironolactone was not explicitly assessed in this study. In a similar investigation by Li et al. [19], the use of potassium-sparing diuretics was associated with a small increase in breast cancer, but again, the use of spironolactone was not specifically evaluated. In contrast, González-Pérez et al. [20] detected no clear association between antihypertensive drugs and breast cancer risk. Subsequent retrospective and longitudinal studies, specifically on spironolactone, also failed to find an association [17,23,25,26,28,36,43,44,45]. Most importantly, a large, matched cohort study of 1.29 million women in the United Kingdom (8.4 patient years) found no association between spironolactone use and breast cancer [25]. Another large cohort study of 2.3 million women (28.8 million person years) in Denmark found no association with neither breast, nor uterine, cervical, or ovarian cancer [26]. Finally, a recent pharmacoepidemiologic study conducted in a real-world context in a large population failed to find a pharmacovigilance signal for spironolactone and breast cancer in women aged ≥50 years when other drugs or pseudo-antialdosterone antagonists were used as comparators [29].

Although several studies could show the good safety profile of spironolactone in the general population, patients with a personal history of breast cancer could be thought at risk of the recurrence of the malignancy when using spironolactone for FAGA [37]. However, in a study of breast cancer survivors, Wei et al. [31], showed that spironolactone was not independently associated with increased breast cancer recurrence and may be considered for the treatment of alopecia in these patients. However, the major limitation of this study is the short follow-up of 2 years [31]. In another study on persistent chemotherapy-induced alopecia (pCIA) in breast cancer survivors, the authors found that despite a hypothetical risk of stimulation of hormone receptor-positive tumors, spironolactone was not associated with an increase in breast cancer recurrence in breast cancer survivors [34]. In addition, spironolactone is used as a cardioprotective agent in breast cancer patients undergoing chemotherapy and it is hypothesized to even inhibit cancer cell growth [46].

In conclusion, based on the large reports published to date, there are insufficient data to link the use of spironolactone to an increased risk of breast and other gynecologic cancers, or an increased risk of breast cancer recurrence [4,25]. As a result, spironolactone may be considered a safe therapy for FAGA and most likely an option for the treatment of alopecia in disease-free breast cancer survivors [31].

## 4. 5α-Reductase Inhibitors (Finasteride/Dutasteride)

5α-reductase inhibitors are primarily used to treat benign prostatic hyperplasia, and finasteride is also used to treat androgenetic alopecia in men. Its use in women is less common because of limited efficacy [47]. Although finasteride is generally safe in women with FAGA based on several studies [4], the use of 5α-reductase inhibitors may induce breast tenderness in men and women, as well as gynecomastia in men; they are thought to increase total serum testosterone levels, leading to increased estrogen levels via aromatization [4]. There are still insufficient data to conclude whether finasteride affects the risk of male breast cancer [48]. However, most of the larger studies fail to find an association between the use of 5α-reductase inhibitors and male breast cancer [49,50,51,52]. Studies evaluating the risk of malignancy, in particular breast cancer, in women with androgenetic alopecia taking finasteride are still lacking [4].

Based on the experience of many years of use in a large number of patients [53], there is currently no evidence that the use of 5α-reductase inhibitors, and finasteride in particular, increases the risk of breast cancer in women. Laboratory results suggest a potential protective effect by negatively regulating in situ DHT production and action, and thus inhibiting cancer cell proliferation, in hormone-dependent breast cancer [54]; therefore, finasteride is postulated to have a protective effect at least in postmenopausal women [55]. However, its use in breast cancer survivors remains controversial without larger trials.

Dutasteride, just like finasteride, is mainly prescribed to men. Recent studies in male patients also suggest that there is no association between the incidence of breast cancer cases and the use of dutasteride [56]. Trials specifically evaluating the safety of dutasteride in women and larger studies in FAGA are still missing [35]. However, dutasteride is routinely used for a cicatricial variant of alopecia, i.e., frontal fibrosing alopecia, without reports of consecutive breast cancer [57,58]. A recent interesting case study evaluated its efficacy in a female patient with estrogen-positive, progesterone-negative, and HER2-negative breast cancer case experiencing endocrine therapy-induced alopecia (EIA) [35]. Dubin et al. [35] observed a significant improvement in hair density shortly after the daily use of 0.5 mg of dutasteride (in combination with topical minoxidil 5%). The patient did not report any notable side effects of dutasteride during her treatment [35].

In summary, the 5α-reductase inhibitors (finasteride/dutasteride) might be an alternative for the treatment of FAGA or even EIA. However, controlled trials are needed to assess their efficacy and their long-term safety in high-risk populations, especially gynecologic cancer survivors.

## 5. Cyproterone Acetate

CPA is a derivative of hydroxyprogesterone, which has both antiandrogenic and some antigonadotropic effects [59]. It is commonly used to treat hirsutism, acne, and androgenetic alopecia in women and is a part of hormonal contraception, hormone replacement therapy, and hormone therapy for transgender women. Breast pain, tenderness, and enlargement, as well as hepatotoxicity, are to be mentioned among others as possible side effects of CPA [59]. Concerns about a possible risk of hepatocellular carcinoma rose after studies in rats; in humans, however, an association with liver cancer and CPA treatment has been refuted [60]. However, recent studies have observed a dose- and exposure-time-dependent association between the use of CPA and the risk of intracranial meningiomas, leading to restrictions on its use in many countries [61]; it remains uncertain whether it is safe below a certain threshold of dose and time [62].

Some studies have demonstrated beneficial effects of CPA in the treatment of advanced male breast cancer and advanced prostate cancer [59,63]. CPA has also been used as part of the treatment for advanced or metastatic breast cancer in postmenopausal women; its use in the treatment of breast cancer has declined over time due to the availability of more effective and targeted therapies [18]. Yet, some studies have suggested a possible association between CPA and an increased risk of breast cancer, especially when used at high doses and for long periods of time. However, these studies were mostly conducted in elderly or transgender patients who were also receiving estrogen as part of their hormone replacement or hormone therapy. The study by Fabre et al. [24], which was not limited to CPA, examined the association between premenopausal use of a progestogen-only product (excluding minipills for contraception) after the age of 40 years and the risk of invasive breast cancer risk in 73,664 women from the French E3N cohort. Although ever using a progestogen before menopause was not significantly associated with risk overall, a significant increase in risk was observed with duration of use, and current use. In this age group, progestogens use for more than 4.5 years was significantly associated with risk. There was no difference between antigonadotropic progestogens including CPA and non-antigonadotropic progestogens [24]. Another review of data from randomized controlled trials and epidemiological studies by Collins et al. on the association between postmenopausal hormone therapy and breast cancer risk found an increase with estrogen–progestin use more than with estrogen alone [21]. In line with this, a nationwide observational study found an association between high-dose CPA plus estrogen and an increased risk of breast cancer in transgender women compared with the incidence in cisgender men. [30]; however, little is known about the cause of the increased risk of breast cancer in transgender women.

In conclusion, CPA may be an optional antiandrogenic treatment for FAGA. However, this therapy is not recommended for patients with known risk factors, such as older age or personal or family history of breast cancer or of meningiomas. Here, also, future controlled trials are needed to evaluate efficacy and long-term safety in younger patients without risk factors who could benefit from this therapy for the treatment of hirsutism, acne, and androgenetic alopecia.

## 6. Flutamide and Bicalutamide

Flutamide is a selective antiandrogen of nonsteroidal structure, without any other hormonal or antihormonal activity, and acts primarily by hindering entry of androgens into the cells by competitively blocking their cytoplasmic and nuclear binding to the receptor [64]. Flutamide is indicated for the treatment of prostate cancer and there has been some off-label use for the treatment of polycystic ovary syndrome, hirsutism, hair loss, and acne. Clinical trials of flutamide in patients with metastatic breast cancer did not show efficacy but were conducted in a patient population that was not selected for hormone receptor status [65]. Possible important side effects of flutamide include liver function abnormalities [64]; it is crucial that all healthcare professionals prescribing flutamide are aware of the black box warning regarding liver failure [66]. A few studies have shown the efficacy of flutamide in the treatment of FAGA at low doses that were considered to be safe [64,67]. This safety has been invalidated in light of fatal outcomes even at such low doses, and it has therefore fallen out of use for this reason [68].

Bicalutamide, a newer and more potent androgen receptor blocker than flutamide, is also approved for the treatment of prostate cancer [69]. A recent study showed that bicalutamide inhibits the proliferation and invasion of triple-negative breast cancer cells by targeting the androgen receptor signaling pathway and down-regulating matrix metalloprotease-2/-9 protein expression levels [70]. Therefore, bicalutamide was also used to treat patients with severe or metastatic breast cancer [27,32]. Bicalutamide has recently been used off-label for FAGA at lower doses [69,71,72]. Mild and transient increases in liver enzymes have been reported as the most frequent adverse effect of oral bicalutamide in the treatment of FAGA [71].

There are still no specific investigations on the treatment of FAGA in breast cancer survivors; however, one interesting study used flutamide (50–75 mg/d) or bicalutamide (10 mg/d) in combination with low-dose oral minoxidil to treat pCIA in breast cancer survivors [34].

Thus, the potent antiandrogens such as flutamide or similar and newer congeners with better pharmacokinetics and safety profiles, such as bicalutamide [66], could be considered as second- or third-line alternatives for the treatment of FAGA resistant to more convenient therapies such as spironolactone. Nevertheless, controlled studies are demanded to assess their safety, especially in high-risk populations.

## 7. Conclusions

Treatment options for FAGA, besides non-specific hair growth stimulators such as minoxidil, are limited to substances interfering with sex hormones to block the androgen pathway implicated in the pathogenesis of FAGA. Androgen receptor blockers, 5-alpha-reductase inhibitors, and oral contraceptives can be considered, although their safe long-term use in patients at risk of or with a history of breast cancer or other gynecological malignancies remains unproven, even though none seem to carry a higher risk of gynecologic malignancy. Yet, several studies have shown reassuring results for spironolactone (Table 3). This could most likely make spironolactone an optimal option in the treatment of FAGA in the high-risk groups. Furthermore, small series of patients with EIA or pCIA treated with the 5α-reductase inhibitors (i.e., finasteride/dutasteride) or potent antiandrogens (i.e., bicalutamide) possibly show their potential use as second- or third-line alternatives for the treatment of FAGA resistant to spironolactone. Future controlled trials are however needed to judge the efficacy and long-term safety of these treatments in patients with FAGA and various risk factors to establish an optimal risk-assessment and treatment-selection protocol.

## Figures and Tables

**Table 1:** Summary of selected drugs in the paper.

Drug Name	Pharmacologic Category	Action Mechanism (Alopecia)
**Spironolactone**	Antihypertensive; potassium-sparing diuretic; aldosterone antagonist	Prevents testosterone and DHT from interacting with intracellular androgen receptors and can weakly inhibit androgen synthesis.
**Dutasteride**	5 Alpha-reductase inhibitor	Prevent the conversion of testosterone to DHT, leaving androgens that do not bind securely to their receptors.
**Finasteride**		
**Cyproterone acetate**	Antiandrogen	Competitively prevents DHT from attaching to its receptors.
**Flutamide**	Antiandrogen, antineoplastic agent	Inhibit the androgen absorption and its nuclear binding in target tissues.
**Bicalutamide**		

**Table 2:** Chronological overview of studies discussed in the text.

No.	Study	Investigated Medication	Main Study Findings	Comments/Remarks
1	Danielson, et al., 1982 [17]	Different medications (including spironolactone)	The results indicate that none of the drugs studied (including spironolactone) had a strong positive association with breast cancer. There was a modest association between breast cancer and recent reserpine use.	
2	Willemse, et al., 1988 [18]	Cyproterone acetate	The anticancerous therapeutic efficacy of CPA in treating advanced breast cancer in postmenopausal patients is disappointing.	Cyproterone acetate as the primary treatment in female breast cancer patients.
3	Li CI, et al., 2003 [19]	Different types of antihypertensive medications	The use of specific antihypertensive medications, such as certain calcium channel blockers and diuretics, might elevate the likelihood of breast cancer among older women. Although ever using diuretics was not associated with breast cancer risk when compared to other antihypertensive medications, the use of both potassium-sparing diuretics and thiazide was linked to a modest increase in breast cancer risk.	The use of spironolactone was not specifically evaluated in this study.
4	González-Pérez, et al., 2004 [20]	Different types of antihypertensive medications	No clear association between the risk of breast cancer and antihypertensive drugs could be found in the study.	
5	Collins, et al., 2005 [21]	Estrogen products with or without progestin	The review of trials of postmenopausal hormone therapy showed that the risk of breast cancer is increased with estrogen–progestin use than with estrogen alone.	The study was not restricted to cyproterone acetate.
6	Largent, et al., 2006 [22]	Different types of antihypertensive medications	This study supports a positive association between breast cancer and treated hypertension in women aged 50–75 years. Diuretic use was associated with elevated breast cancer risk. This risk increased with the duration of use. Use of other blood pressure medications was not found to be associated with breast cancer risk.	The use of spironolactone was not explicitly assessed in this study.
7	Fryzek, et al., 2006 [23]	Different types of antihypertensive medications	There was no statistically significant association between breast cancer and ever using any antihypertensive medication overall or any specific class of antihypertensive medication (diuretics, calcium channel blockers, beta-blockers, angiotensin II antagonists, and angiotensin-converting enzyme inhibitors).	
8	Fabre, et al., 2007 [24]	Oral progestagen-only products (except for contraceptive mini-pills)	Prolonged use of oral progestins (without estrogens) prior to menopause by women over the age of 40 may increase the risk of breast cancer.	The study was not restricted to cyproterone acetate.
9	Mackenzie, et al., BMJ. 2012 [25]	Spironolactone	The study suggests that long-term treatment with spironolactone for cardiovascular disease does not increase the risk of breast cancer in women older than 55 years with no history of the disease.	The study included approximately 1.3 million patients with a total follow-up of 8.4 million patient years.
10	Biggar, et al., 2013 [26]	Spironolactone	There is no evidence of increased risk of breast, uterine, ovarian, or cervical cancer with the use of spironolactone or furosemide.	The study assessed cancer incidence in 2.3 million women with a follow-up of 28.8 million person years.
11	Gucalp, et al., 2013 [27]	Bicalutamide	The clinical benefit rate of 19% observed with bicalutamide provides proof of principle of the efficacy of minimally toxic androgen blockade in a select group of patients with estrogen receptor-/progesterone receptor-negative, androgen receptor-positive breast cancer.	Bicalutamide for treatment of severe or metastatic breast cancer.
12	Mackenzie, et al., 2017 [28]	Spironolactone	There was no evidence of an increased risk of any cancer associated with spironolactone use.	Significantly lower risk of prostate cancer associated with spironolactone use.
13	Sabatier, et al., 2019 [29]	Spironolactone	This study found no evidence of breast cancer associated with spironolactone.	
14	Rozner, et al., 2019 [4]	Spironolactone	Most patients did not show increased estrogen levels with spironolactone. There were no data suggesting an increased risk of breast cancer.	
15	de Blok, et al., 2019 [30]	Combination of antiandrogens and estrogens. Antiandrogen treatment usually consisted of cyproterone acetate or spironolactone	The risk of breast cancer increased during a relatively short period of hormone treatment in trans women. The characteristics of the breast cancer resembled a more female pattern.	The reasons for the increased risk of breast cancer in transgender women remain largely elusive.
16	Wei, et al., 2020 [31]	Spironolactone	Spironolactone was not independently associated with increased breast cancer recurrence and might therefore be considered to treat alopecia in breast cancer survivors.	
17	Lu, et al., 2020 [32]	Bicalutamide orally plus another aromatase inhibitor	Combining bicalutamide with another aromatase inhibitor did not demonstrate synergistic activity in patients with estrogen receptor-positive breast cancer who were resistant to aromatase inhibitors.	Bicalutamide for treatment of severe or metastatic breast cancer.
18	Bommareddy, et al., 2020 [33]	Spironolactone	No association between a substantial increased risk of cancer and the use of spironolactone could be observed in this systematic review.	
19	Bhoyrul, et al., 2021 [34]	Minoxidil combined with an antiandrogen	The study used low-dose oral minoxidil in combination with an antiandrogen drug (spironolactone (25–100 mg/d), bicalutamide (10 mg/d), or flutamide (50–75 mg/d)) to treat pCIA ^#^ in a series of breast cancer survivors.	
20	Dubin, et al., 2023 [35]	Dutasteride	The study showed a significant improvement in hair density shortly after daily use of 0.5 mg of dutasteride (in combination with topical minoxidil 5%) in one case of EIA *.	
21	Hill, et al., 2024 [36]	Spironolactone	The study found no evidence of an association between spironolactone exposure for dermatologic conditions and the risk of uterine or breast tumors compared with unexposed women.	

* EIA: endocrine therapy-induced alopecia; ^#^ pCIA: chemotherapy-induced alopecia.

**Table 3:** Suggested decision algorithm for the treatment of female androgenetic alopecia following gynecologic malignancies, based on the available guidelines and studies.

Suggested Decision Algorithm for the Treatment of Female Androgenetic Alopecia Following Gynecologic Malignancies
1st line: Minoxidil topical, or possibly systemic minoxidil *
2nd line: Spironolactone *
3rd line (refractory or very sever cases): 5α-reductase inhibitors (i.e., finasteride/dutasteride) *^#^ or potent antiandrogens (i.e., bicalutamide) *^#^

* Off-label in many countries; ^#^ their use in high-risk populations, particularly gynecologic cancer survivors, remains controversial in the absence of larger trials.

## Data Availability

The data supporting the results of this study are presented in the current paper.
